# The Recognition of YopJ Family Effectors Depends on ZAR1/JIM2 Immune Complex in *Nicotiana benthamiana*

**DOI:** 10.1111/mpp.70214

**Published:** 2026-02-05

**Authors:** Injae Kim, Jieun Kim, Ye Jin Ahn, Kee Hoon Sohn, Cécile Segonzac

**Affiliations:** ^1^ Department of Agriculture, Forestry and Bioresources Seoul National University Seoul Republic of Korea; ^2^ Global Plant Immunity Research Center Seoul National University Seoul Republic of Korea; ^3^ Department of Agricultural Biotechnology Seoul National University Seoul Republic of Korea; ^4^ Plant Genomics and Breeding Institute Seoul National University Seoul Republic of Korea; ^5^ Plant Health Center Seoul National University Seoul Republic of Korea; ^6^ Research Institute of Agriculture and Life Sciences Seoul National University Seoul Republic of Korea

**Keywords:** convergent evolution, nucleotide‐binding leucine‐rich repeat receptor, plant immune system, type III secreted effector

## Abstract

Pathogens deploy effector proteins to manipulate host physiology and promote infection. YopJ family effectors are highly conserved across bacterial genera that cause crop diseases. Nucleotide‐binding leucine‐rich repeat receptors (NLRs) play a central role in direct or indirect recognition of effectors and trigger immune responses, including hypersensitive cell death (HR). Two NLRs, *Nicotiana benthamiana* homologues of Pseudomonas tomato race 1 (NbPtr1) and HOPZ‐ACTIVATED RESISTANCE 1 (NbZAR1), were recently identified as independently recognising two YopJ family effectors, HopZ5 and AvrBsT/XopJ2. NbZAR1 also detects XopJ4 via the receptor‐like cytoplasmic kinase XOPJ4 IMMUNITY 2 (JIM2). Here, we conducted *Agrobacterium*‐mediated transient expression assays with 20 YopJ family effectors from five phytopathogenic bacterial genera and identified 12 YopJ family effectors that are recognised either by NbZAR1 or independently by NbZAR1 and NbPtr1. Furthermore, we show that YopJ family effector‐induced HR is differentially suppressed by the deacetylase SUPPRESSOR OF AVRBST‐ELICITED RESISTANCE 1, suggesting more than one mechanism for YopJ family effector recognition. This work provides the genetic basis of the recognition of YopJ family effectors in *N. benthamiana* and lays a foundation for the mechanistic study of NbZAR1/JIM2 and NbPtr1 mode of activation.

## Graphical abstract

The cell death triggered by different YopJ family effectors in *Nicotiana benthamiana* results from NbZAR1 and/or NbPtr1 activation and is differentially suppressed by the deacetylase SOBER1.

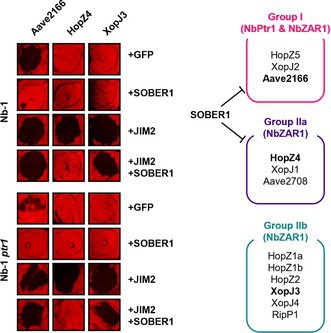

Significant yield losses in a wide range of crop species are due to bacterial diseases (Savary et al. 2019). The type III secretion system (T3SS) is a major virulence determinant of phytopathogenic bacteria. This syringe‐like apparatus enables direct translocation of type III effector proteins (T3Es) into host cells (Coll and Valls 2013). Once delivered, T3Es manipulate host physiology by targeting and modifying specific host proteins (Landry et al. 2020; Peeters et al. 2013; Sabbagh et al. 2019). The Yersinia outer protein J (YopJ) family represents one of the most widely distributed groups of T3Es, present in *Pseudomonas syringae* , *Xanthomonas* spp., *Ralstonia solanacearum* , *Erwinia amylovora* and *Acidovorax citrulli* (Lewis et al. 2011; Ma and Ma 2016). YopJ family effectors belong to the C55 protease family, characterised by conserved catalytic residues (Orth et al. 2000). Given the mechanistic similarities between cysteine proteases and acetyltransferases, some YopJ effectors exhibit bifunctional enzymatic activities (Cheong et al. 2014; Ma et al. 2006; Orth et al. 2000). Phytopathogenic bacteria employ YopJ family effectors to enhance virulence in host plants. The *P. syringae* effector HopZ1a disrupts the host microtubule network by acetylating tubulin, inhibiting secretion of antimicrobials (Lee et al. 2012). The *Xanthomonas euvesicatoria* effector AvrBsT/XopJ2 suppresses AvrBs1‐triggered cell death and acetylates ACETYLATED INTERACTING PROTEIN1, a microtubule‐associated protein involved in antibacterial immunity (Cheong et al. 2014; Szczesny et al. 2010). Other YopJ family members, such as HopZ4 and XopJ1, interfere with host protein secretion pathways (Üstün et al. 2013, 2014). Additionally, nuclear‐localised YopJ homologues such as XopJ6 and RipP2/PopP2 acetylate WRKY transcription factors to suppress plant defence responses (Lauber et al. 2024; Le Roux et al. 2015; Sarris et al. 2015). Immunosuppressive functions of YopJ family effectors likely underlie the broad conservation of these virulence factors in pathogenic bacteria.

Plants can indirectly detect pathogenic bacteria by monitoring chemical modifications of T3E targets. Modifications of the so‐called host guardee/decoy activate nucleotide‐binding leucine‐rich repeat receptors (NLRs) and elicit calcium‐dependent immune responses, including reactive oxygen species (ROS) accumulation, phospho‐relay and transcriptional reprogramming (Kim, Ahn, et al. 2023; Ngou et al. 2022). NLR‐triggered immunity often culminates in the hypersensitive response (HR), a form of rapid programmed cell death that can limit pathogen spread (Bi et al. 2021; Gao et al. 2013). The *Arabidopsis* coiled‐coil NLR (CNL) HOPZ‐ACTIVATED RESISTANCE 1 (ZAR1) is one of the best characterised plant NLRs and participates in the indirect recognition of diverse T3Es (Laflamme et al. 2020; Lewis et al. 2010). ZAR1 monitors modifications of receptor‐like cytoplasmic kinases (RLCKs) to detect effector activity (Ma et al. 2006; Martel et al. 2020; Seto et al. 2017; Wang et al. 2019). In *Arabidopsis*, the *P. syringae* effector HopZ1a directly acetylates the RLCK‐XII pseudokinase HopZ‐ETI‐Deficient1, which interacts with ZAR1 and triggers immune activation (Lewis et al. 2013). Similarly, the *Nicotiana benthamiana* homologue of ZAR1 (NbZAR1) recognises XopJ1, XopJ3, XopJ4 and RipP1 effectors via the RLCK‐XII protein XOPJ4 IMMUNITY 2 (JIM2) (Schultink et al. 2019; Staskawicz and Schultink 2024). NbZAR1 also detects the *X. euvesicatoria* effector AvrBsT/XopJ2 and the *P. syringae* pv. *actinidiae* effector HopZ5 (Ahn et al. 2023). Both HopZ5 and AvrBsT/XopJ2 are also independently recognised by NbPtr1, the *N. benthamiana* homologue of Pseudomonas tomato race 1 resistance protein, revealing that individual T3Es can be perceived by non‐orthologous NLRs within plant species (Ahn et al. 2023; Kim, Ahn, et al. 2023; Mazo‐Molina et al. 2020).

Previous studies reported that other YopJ family effectors can induce cell death in *N. benthamiana* (Figure S1) (Albers et al. 2019; Ma et al. 2006; Thieme et al. 2007; Traore et al. 2019; Vinatzer et al. 2006). Here, we used *Agrobacterium* to transiently express 20 representative YopJ family T3Es in *N. benthamiana* plants that are knocked down or knocked out for *NbZAR1* and/or *NbPtr1* to demonstrate the genetic basis of YopJ family T3E recognition. We found that 12 YopJ family T3Es are indirectly recognised through their predicted enzymatic activity. Additionally, we report differential suppression of NbZAR1/JIM2‐ and NbPtr1‐mediated HR by the deacetylase SUPPRESSOR OF AVRBST ELICITED RESISTANCE 1 (SOBER1) (Bürger et al. 2017; Choi et al. 2021; Cunnac et al. 2007). The specificity of YopJ family T3E recognition by NbZAR1 and/or NbPtr1 aligns with phylogenetic grouping, providing mechanistic insights for the mode of activation of two immune receptors, which could aid in crop protection against diverse bacterial diseases.

We selected 20 YopJ family T3Es based on annotations in the T3E repertoires of *P. syringae* , *Xanthomonas* spp., *R. solanacearum* , *A. citrulli* and *E. amylovora* (Figure S1) (Ahn et al. 2023; Lauber et al. 2024; Ma and Ma 2016; Peeters et al. 2013). The corresponding sequences harbour predicted catalytic residues characteristic of the YopJ family and cofactor inositol hexaphosphate (InsP6)‐binding site, except RipJ and HopZ1c, respectively (Figure 1a) (Ma et al. 2015; Pandey et al. 2021; Xia et al. 2021; Zhang et al. 2016; Zhou et al. 2009). The RLCK‐XII family protein, JIM2, is essential for NbZAR1 activation and is expressed at a low level in *N. benthamiana* (Figure S2) (Kurotani et al. 2025). In *N. benthamiana* Nb‐1 accession, *JIM2* expression is undetectable and transient expression of JIM2 is required to enable NbZAR1‐mediated cell death (Ahn et al. 2023; Schultink et al. 2019). Here, we conducted experiments in the Nb‐1 accession to evaluate the contribution of the NbZAR1/JIM2 complex in YopJ effector recognition. We monitored the cell death induced by YopJ family T3Es co‐expressed with JIM2 or GFP as a control. We used XopAU, an unrelated *X. euvesicatoria* T3E, as a positive control for cell death (Figure 1b) (Teper et al. 2018). Cell death intensity was quantified by chlorophyll fluorescence measurements, expressed as the quantum yield (QY) of the photosystem II (the ratio of the chlorophyll maximal [Fm] and variable [Fv] fluorescence following a saturating light pulse in dark‐adapted tissues). Low QY values reflect cell death in the agro‐infiltrated tissues (Figure S3) (Kim, Kim, et al. 2023). In Nb‐1 TRV:EV (empty vector) plants, at 3 days post‐infiltration, we observed three categories of responses. Aave2166, AvrBsT/XopJ2 and HopZ5 form a first group (group I) that triggered a robust cell death when co‐expressed with GFP, regardless of additional expression of JIM2. A second group (group II) comprising HopZ1a, HopZ1b, HopZ2, HopZ4, XopJ1, XopJ3, XopJ4, RipP1 and Aave2708 induced cell death only when co‐expressed with JIM2. Lastly, HopZ1c, HopZ3, XopJ5, XopJ6, RipJ, RipK, RipP2 and Eop1 caused weak or no cell death regardless of JIM2 expression and were categorised into group III (Figures 1b and Figure S3). As we confirmed the accumulation of all the YopJ family T3E proteins by immunoblotting (Figure S4), the lack of response in tissues expressing group III T3Es can be interpreted as a lack of recognition in *N. benthamiana*.

**FIGURE 1:**
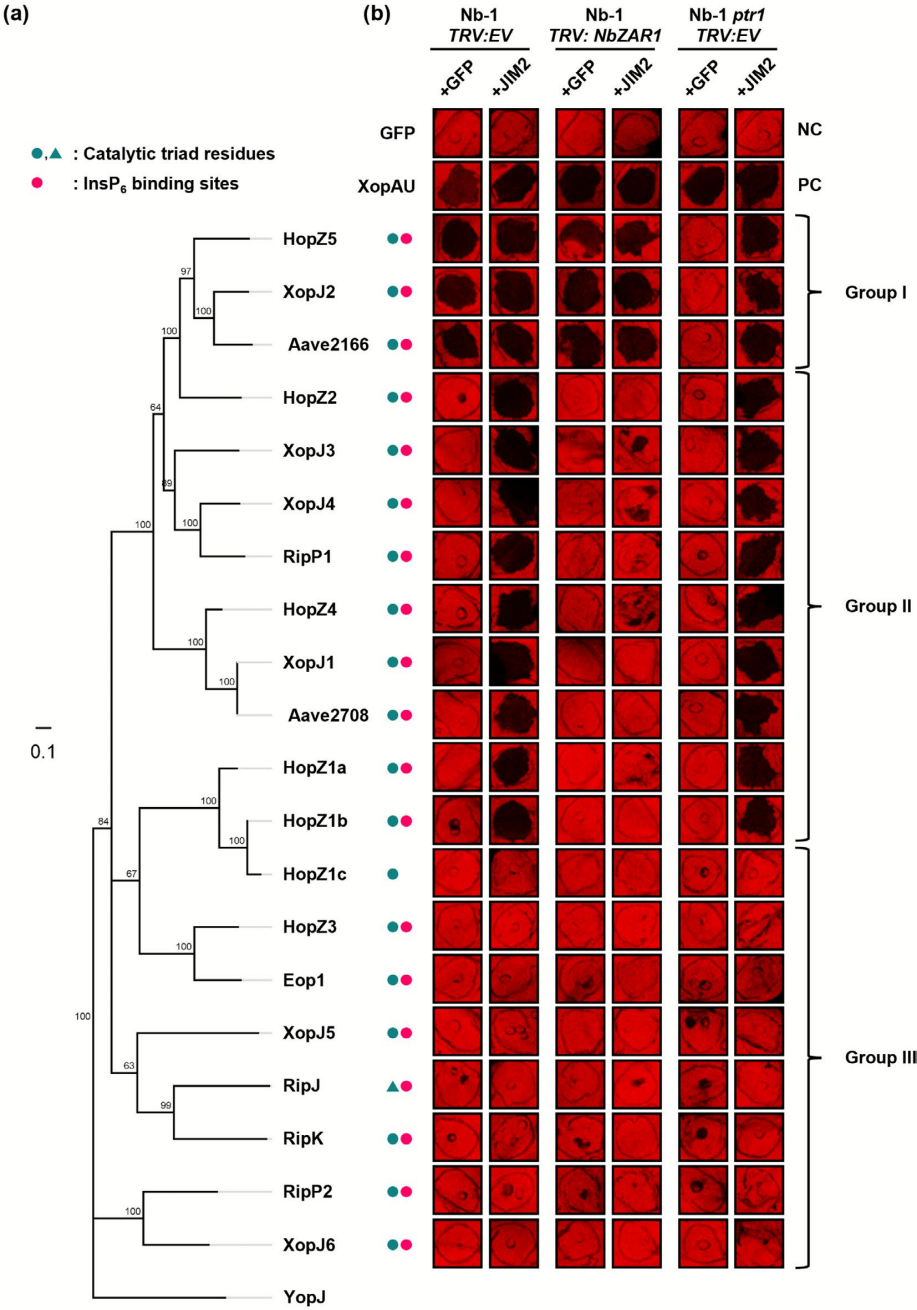
Twelve YopJ family effectors trigger NbPtr1‐ and/or NbZAR1/JIM2‐dependent cell death in *Nicotiana benthamiana*. (a) Phylogenetic tree of 21 YopJ family effectors. The consensus tree was constructed based on multiple sequence alignment using Clustal Omega v. 1.2.2 with bootstrap resampling. Genetic distances were calculated using the Jukes‐Cantor model, and the tree was built using the neighbour‐joining method. *Yersinia* YopJ was used as an outgroup from an animal pathogen. Green circles and triangles indicate conserved catalytic residues (cysteine‐based and serine‐based, respectively). Pink circles denote the presence of InsP_6_ binding sites (Zhang et al. 2016). (b) Green fluorescent protein (GFP), XopAU and each YopJ family effector C‐terminally fused with yellow fluorescent protein (YFP) (OD_600_ = 0.5) were co‐expressed with P19 (OD_600_ = 0.1) and either GFP (OD_600_ = 0.5) or JIM2‐4xMyc (OD_600_ = 0.5) in *N. benthamiana* wild‐type plants (Nb‐1) silenced for empty vector (*TRV:EV*) or *NbZAR1* (*TRV:NbZAR1*) or in *ptr1* knock‐out plants (Nb‐1 *ptr1 TRV:EV*). NC indicates a negative control, and PC indicates a positive control of the experiment. Infiltrations were photographed in light‐emitting diode (LED) light (red‐orange 617 nm and cool white 6500 K) at 3 days post‐infiltration (dpi). The cell death was quantified by measuring chlorophyll quantum yield (QY) (Figure S3). This experiment was independently repeated twice with at least three replicated spots. Group I effectors are recognised by both NbPtr1 and NbZAR1. Group II effectors are recognised by NbZAR1 only. Group III effectors do not trigger cell death.

In the presence of JIM2, the cell death induced by group II T3Es was lost in Nb‐1 plants silenced for *NbZAR1* (Figures 1b and Figure S3). Hence, our analysis demonstrates that 12 out of the selected 20 YopJ family effectors, including T3Es that are previously reported to trigger cell death in *N. benthamiana*, are recognised by NbZAR1/JIM2 (Ahn et al. 2023; An et al. 2025; Ma et al. 2006; Schultink et al. 2019). Furthermore, because XopJ2 and HopZ5 (group I) are independently recognised by NbPtr1 (Ahn et al. 2023), we expressed the 20 YopJ family T3Es in Nb‐1 *ptr1*, a *NbPtr1* knock‐out line generated using CRISPR/Cas9 technology (Ahn et al. 2023). HopZ5, XopJ2 and Aave2166 (Group I) co‐expressed with GFP could not induce cell death in Nb‐1 *ptr1*, but co‐expression with JIM2 restored intense cell death (Figures 1b and Figure S3). Additionally, we confirmed that group I YopJ T3Es failed to induce cell death in Nb‐1 *ptr1* TRV:*NbZAR1* plants (Figures 1b and Figure S5). Thus, Aave2166 from *A. citrulli* , AvrBsT/XopJ2 from *X. euvesicatoria* and HopZ5 from *P. syringae* are independently recognised by NbZAR1 and NbPtr1. Using complementation assays with silencing‐proof synthetic NbZAR1 and NbPtr1, we confirmed that these NLRs are genetically required for the recognition of YopJ family effectors (Figure S6). Notably, the YopJ family effectors recognised by NbZAR1 and/or NbPtr1 are phylogenetically close to one another, and group I effectors form a subclade, indicating that YopJ effector recognition in *N. benthamiana* depends on conserved activity or common host guardees/decoys that the effectors target.

NLRs can sense the presence of effectors through monitoring effector activities (Jones et al. 2024). The 20 selected YopJ effectors harbour a conserved catalytic triad including cysteine, histidine, and either aspartate or glutamate residues, with the exception of RipJ (Figure 1a) (Pandey et al. 2021). To assess the role of the T3E enzymatic activity for the activation of NbZAR1/JIM2 and NbPtr1, we generated cysteine‐to‐alanine substitution (C/A) mutants for the three effectors from group I and the nine effectors from group II that induce NLR‐dependent cell death. This substitution of the conserved cysteine was shown to impair the catalytic activity and the recognition of HopZ1a and RipP2 (Lee et al. 2012; Tasset et al. 2010). The 12 C/A mutants induced significantly reduced or no cell death compared to their wild‐type counterparts in Nb‐1 plants (Figure 2 and Figure S7). Because all C/A mutants accumulated to detectable protein levels (Figure S8), these results indicate that the predicted catalytic activity of the group I and group II effectors is required for the recognition by NbZAR1 and/or NbPtr1, consistent with the previously established indirect mode of activation of these NLRs (Ahn et al. 2023; Mazo‐Molina et al. 2020; Schultink et al. 2019).

**FIGURE 2:**
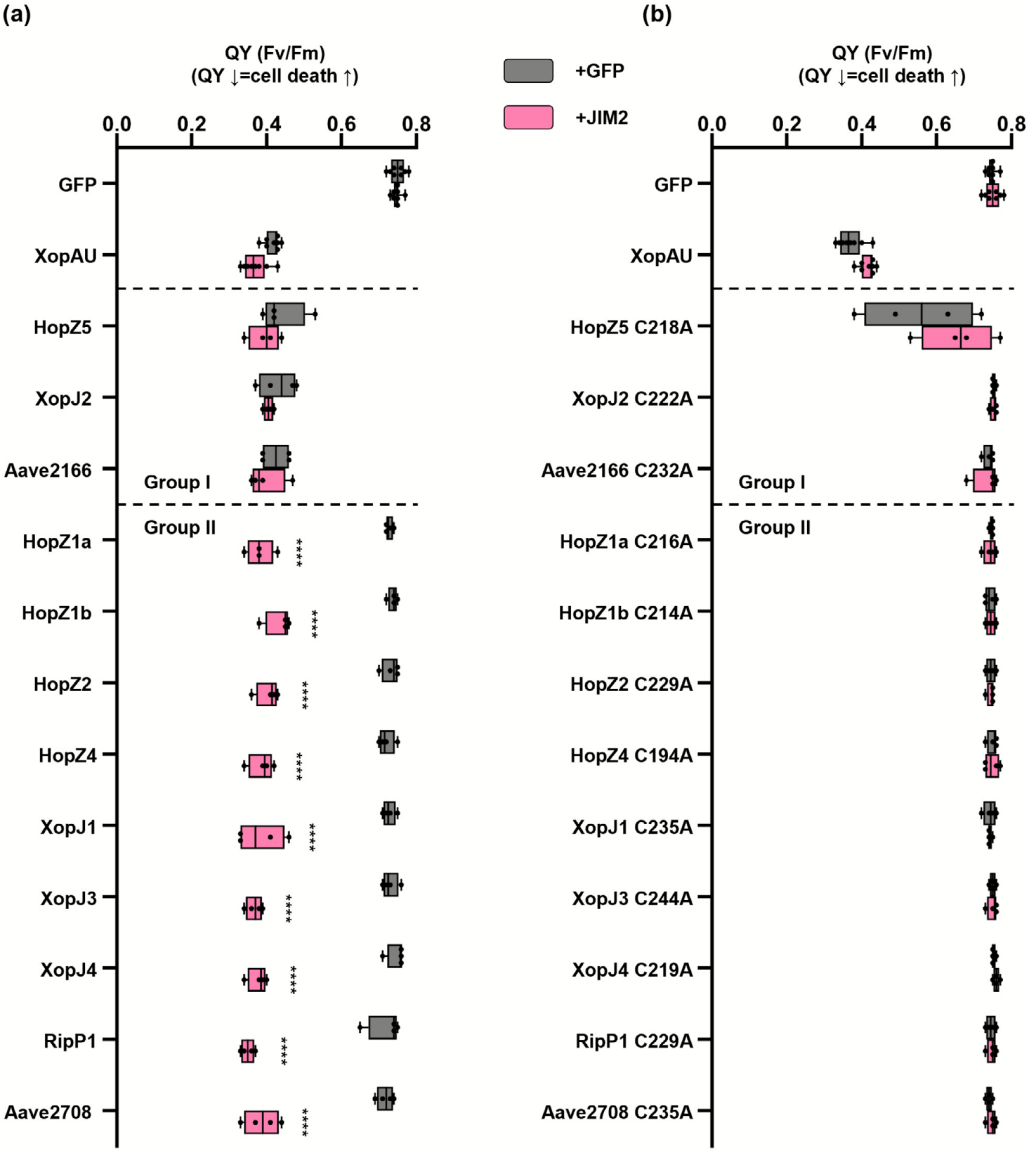
The conserved catalytic cysteine of YopJ family effectors is required for recognition by NbPtr1 and NbZAR1. GFP, XopAU and YopJ family effectors (a) or their corresponding catalytic cysteine mutants (C/A) (b) were co‐expressed with P19 (OD_600_ = 0.1) and with GFP (OD_600_ = 0.5) or JIM2‐4×Myc (OD_600_ = 0.5) in *Nicotiana benthamiana* wild‐type plants (Nb‐1). Infiltrated leaves were photographed in light‐emitting diode (LED) light at 3 days post‐infiltration (Figure S7). Cell death intensity was quantified by measuring the quantum yield (QY) of each infiltrated spot. High QY indicates strong cell death at the infiltration site, and low QY indicates weak or no cell death. Box plots show the distribution of individual values between the lower and upper quartiles (25%–75%), individual values (dots) and median value (line). The whiskers indicate minimum and maximum values. Statistical analysis was performed using two‐way ANOVA, with JIM2 co‐expression as the main factor and effector identity included as a blocking factor to account for grouped comparisons. Dunnett's multiple comparison test was applied post hoc. Asterisks indicate statistically significant differences between the co‐expression of GFP and JIM2 (****p < 0.0001). This experiment was independently repeated twice.

While a protease activity has been reported at low levels for some YopJ family effectors (Ma et al. 2006; Üstün et al. 2014; Üstün and Börnke 2015), subsequent studies reported that YopJ family effectors predominantly modify their host targets via acetylation (Cheong et al. 2014; Choi et al. 2021; Jeleńska et al. 2021; Le Roux et al. 2015; Lee et al. 2012; Rufián et al. 2021). To further assess the relationship between the acetyltransferase activity of YopJ family effectors and their recognition by NbPtr1 and NbZAR1, we co‐expressed the 12 recognised YopJ family effectors with the deacetylase SOBER1 in Nb‐1 and Nb‐1 *ptr1* plants (Figure 3 and Figure S9). In Nb‐1 plants, in the presence of JIM2, the cell death induced by HopZ4, XopJ1 and Aave2708 (group IIa) was completely suppressed by SOBER1, while the cell death induced by HopZ1a, HopZ1b, HopZ2, XopJ3, XopJ4 and RipP1 (group IIb) was not. Furthermore, the NbPtr1‐dependent cell death triggered by HopZ5, XopJ2 and Aave2166 observed in Nb‐1 plants in the presence of GFP was significantly suppressed by SOBER1 (Figure 3 and Figure S9). However, SOBER1 did not suppress the NbZAR1‐dependent recognition of these three group I effectors, as observed in Nb‐*ptr1* plants co‐expressing JIM2. The effector protein accumulation was unaffected by the presence of SOBER1 (Figure S10). These observations are consistent with previous reports showing that SOBER1 displays substrate selectivity among YopJ family effectors in *Nicotiana tabacum* , suppressing XopJ2/AvrBsT and HopZ5 but not HopZ1a (Cheong et al. 2014; Choi et al. 2018, 2021; Jayaraman et al. 2017). Although early studies suggested that YopJ proteins may exhibit low protease activity, accumulating biochemical and structural evidence indicates that YopJ family effectors primarily function as acetyltransferases that modify host signalling components. Group IIb effectors may act as proteases whose activity cannot be reversed by SOBER1. However, we did not observe any change in JIM2 abundance or apparent molecular weight in the presence of either group IIa or group IIb effectors (Figure S8), making proteolysis of JIM2 unlikely. Our finding that SOBER1 suppresses only a subset of YopJ‐triggered cell death is therefore more plausibly explained by variations in effector‐dependent acetylation rather than by distinct enzymatic activities. One possibility is that group II effectors acetylate partially overlapping but distinct sets of host proteins, with JIM2 and NbZAR1 guarding acetylation events likely occurring on additional intermediates, such as members of the RLCKVII family. In this regard, SOBER1 may selectively deacetylate only a subset of these intermediates. Alternatively, different YopJ proteins may target distinct residues or structural contexts on substrates: group IIa effectors may modify SOBER1‐sensitive sites, whereas group IIb effectors may acetylate residues distributed across multiple positions inaccessible for SOBER1. Quantitative differences in acetylation may further contribute, whereby stronger or more persistent acetylation could exceed the deacetylase capacity of SOBER1.

**FIGURE 3:**
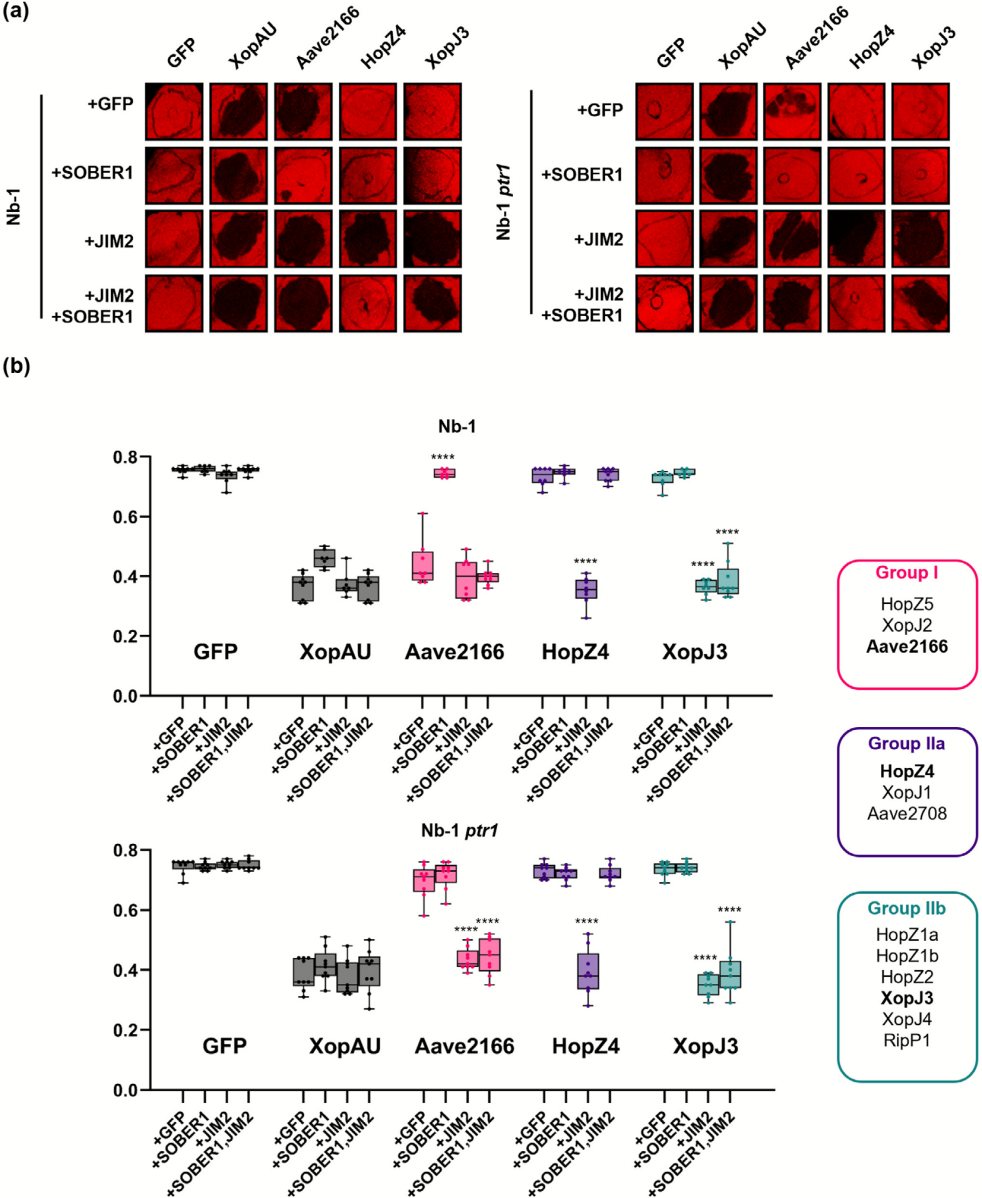
Both NbPtr1‐ and NbZAR1‐dependent recognition of YopJ family effectors can be suppressed by the deacetylase SOBER1. (a) GFP, XopAU, Aave2166 (representative of group I), HopZ4 (representative of group II a) and XopJ3 (representative of group IIb) (OD_600_ = 0.1) were co‐expressed with P19 (OD_600_ = 0.1), and with GFP (OD_600_ = 0.4), with SOBER1‐FLAG (OD_600_ = 0.4) and GFP (OD_600_ = 0.1), with JIM2‐4×Myc (OD_600_ = 0.1) and GFP (OD_600_ = 0.4), or with JIM2‐4×Myc (OD_600_ = 0.1) and SOBER1‐FLAG (OD_600_ = 0.4) in *Nicotiana benthamiana* wild‐type plants (Nb‐1) and Nb‐1 *ptr1* plants. Infiltrated leaves were photographed in light‐emitting diode (LED) light at 3 days post‐infiltration (dpi). (b) The cell death was quantified by measuring quantum yield (QY) at 3 dpi. High QY indicates strong cell death at the infiltration site, and low QY indicates weak or no cell death. Box plots show the distribution of individual values between the lower and upper quartiles (25%–75%), individual values (dots) and the median (line). The whiskers indicate the minimum and maximum values. Statistical analysis was performed using two‐way ANOVA, with the co‐expressed protein as the main factor and effector identity included as a blocking factor to account for grouped comparisons. Dunnett's multiple comparison test was applied post hoc. Asterisks indicate statistically significant differences compared to GFP co‐expression for each effector (****p < 0.0001). These experiments were independently repeated twice, with at least four replicated spots per condition. Boxes indicate the phenotypic effector groups; data for each of the 12 type III effector proteins are presented in Figure S9.

In summary, our cell death assays demonstrate that NbZAR1 can recognise 12 YopJ family T3Es in the presence of the key immune component JIM2, providing a genetic basis for the broad recognition spectrum of NbZAR1 and supporting convergently evolved effector recognition by NbZAR1 and NbPtr1 (Ahn et al. 2023; Kim, Ahn, et al. 2023). The differential suppression by SOBER1 among YopJ family effectors further indicates that NbPtr1 and NbZAR1 rely on distinct recognition modes, with NbZAR1 likely acting together with JIM2 and additional guardee/decoy proteins.

## Author Contributions

**Cécile Segonzac:** conceptualization, funding acquisition, writing – original draft, writing – review and editing, supervision, resources. **Kee Hoon Sohn:** conceptualization, resources, supervision. **Injae Kim:** conceptualization, investigation, analysis, writing – original draft. **Jieun Kim:** investigation, analysis, methodology. **Ye Jin Ahn:** methodology, writing – review and editing.

## Funding

This work was supported by the National Research Foundation of Korea (RS‐2018‐NR031006 and RS‐2024‐00349151) and Korea Institute of Planning and Evaluation for Technology in Food, Agriculture, Forestry and Fisheries (RS‐2024‐00398300).

## Conflicts of Interest

The authors declare no conflicts of interest.

## Supporting information


**Figure S1:** Features of the 20 YopJ family effectors selected for this study.

**Figure S2:** Gene expression profile of *JIM2*, *NbZAR1* and *NbPtr1*. Expression of *JIM2*, *NbZAR1* and *NbPtr1* in transcript per million (TPM) was retrieved on the NbenBase gene expression tool (https://nbenthamiana.jp/nbrowser/profile) (Kurotani et al. 2025). Red arrows indicate leaves used for the agro‐infiltration experiments.

**Figure S3:** Quantification of cell death in YopJ family T3E expressing tissues shown in Figure 1a. Cell death intensity was quantified by measuring the quantum yield (QY) of each infiltrated spot. High QY indicates strong cell death at the infiltration site, and low QY indicates weak or no cell death. Box plots show the distribution of individual values between the lower and upper quartiles (25%–75%), individual values (dots) and median value (line). The whiskers indicate minimum and maximum values. Statistical comparisons were performed using two‐way ANOVA with JIM2 co‐expression as a main factor and effector identity included as a blocking factor to account for grouped comparisons, followed by Dunnett's multiple comparison test. Asterisks indicate statistically significant differences compared with the GFP control co‐expressed with GFP or JIM2 for each effector in each type of plant (**p < 0.01; ***p < 0.001; ****p < 0.0001). This experiment was independently repeated twice with at least four replicated spots.

**Figure S4:** Accumulation of YopJ family T3E proteins in tissues shown in Figure 1. GFP and effector‐YFP fusions were expressed with JIM2‐4xMyc in Nb‐1 *ptr1 TRV: NbZAR1* tissues. Leaf samples were harvested 40–60 h after agroinfiltration. Immunodetection on total protein extracts was performed with anti‐GFP antibodies. Ponceau S staining (PS) shows equal loading of the samples.

**Figure S5:** HopZ5, XopJ2 and Aave2166 are independently recognised by NbPtr1 and NbZAR1. (a) GFP, XopAU and each YopJ family effector were co‐expressed with P19 and either GFP or JIM2‐4xMyc in Nb‐1 *TRV: EV* or in Nb‐1 *ptr1 TRV: NbZAR1* silenced plants. (b) Cell death intensity was quantified by measuring the quantum yield (QY) of each infiltrated spot. High QY indicates strong cell death at the infiltration site, and low QY indicates weak or no cell death. Box plots show the distribution of individual values between the lower and upper quartiles (25%–75%), individual values (dots) and median value (line). The whiskers indicate minimum and maximum values. Statistical comparisons were performed using two‐way ANOVA with JIM2 co‐expression and types of plant as main factors and effector identity included as a blocking factor to account for grouped comparisons, followed by Dunnett's multiple comparison test. Asterisks indicate statistically significant differences between Nb‐1 *TRV: EV* and Nb‐1 *ptr1 TRV: NbZAR1* for each effector (****p < 0.0001). This experiment was independently repeated twice with at least 4 replicated spots.

**Figure S6:** Transient expression of synthetic NbPtr1 and NbZAR1 complements the recognition of YopJ family T3Es in *NbPtr1* knock‐out and *NbZAR1* knock‐down plants. Cell death intensity was quantified by measuring the quantum yield (QY) of each infiltrated spot. High QY indicates strong cell death at the infiltration site, and low QY indicates weak or no cell death. Box plots show the distribution of individual values between the lower and upper quartiles (25%–75%), individual values (dots) and median value (line). The whiskers indicate minimum and maximum values. Statistical comparisons were performed using two‐way ANOVA with JIM2 co‐expression as a main factor and effector identity included as a blocking factor to account for grouped comparisons, followed by Dunnett's multiple comparison test. These experiments were independently repeated twice with at least four replicated spots. (a) GFP and group I YopJ family effectors (OD_600_ = 0.5) were co‐expressed with P19 (OD_600_ = 0.1) and either GFP or synNbPtr1‐6xHA (OD_600_ = 0.05) in Nb‐1 *ptr1* plants. Infiltrated leaves were photographed in light‐emitting diode (LED) light at 3 dpi. Asterisks indicate statistically significant differences between co‐expressed with GFP or synNbPtr1‐6XHA for each effector in each type of plant (**p < 0.01; ****p < 0.0001). (b) GFP, group I and II YopJ family effectors (OD_600_ = 0.5) were co‐expressed with P19 (OD_600_ = 0.1), JIM2‐4XMYC (OD_600_ = 0.05) and either GFP or synNbZAR1‐4XMYC (OD_600_ = 0.05) in Nb‐1 *ptr1 TRV: EV* and *TRV: NbZAR1* silenced plants. Infiltrated leaves were photographed in light‐emitting diode (LED) light at 2 dpi. Asterisks indicate statistically significant differences of JIM2‐mediated cell death between co‐expression with GFP or synNbZAR1‐4XMYC for each effector. (*p < 0.05; ***p < 0.001; ****p < 0.0001).

**Figure S7:** Photographs of infiltrated leaf spots shown in Figure 2. Leaves were photographed in light‐emitting diode (LED) light (red‐orange 617 nm and cool white 6500 K) at 3 dpi.

**Figure S8:** Accumulation of YopJ family T3E C/A mutant proteins in tissues shown in Figure 2. GFP and C/A mutant‐YFP fusions were expressed with JIM2‐4xMyc in Nb‐1 *ptr1 TRV: NbZAR1* tissues. Leaf samples were harvested 40–60 h after agroinfiltration. Immunodetection was performed with anti‐GFP and anti‐MYC antibodies. Ponceau S staining (PS) shows equal loading of the samples.

**Figure S9:** Photographs of infiltrated leaf spots shown in Figure 3. Leaves were photographed in light‐emitting diode (LED) light (red‐orange 617 nm and cool white 6500 K) at 3 dpi.

**Figure S10:** Accumulation of YopJ family T3E, JIM2 and SOBER1 proteins in tissues shown in Figure 3. GFP and effector‐YFP fusions were expressed with JIM2‐4xMyc and SOBER1‐3xFLAG in Nb‐1 *ptr1 TRV: NbZAR1* tissues. Leaf samples were harvested 40–60 h after agroinfiltration. Immunodetection was performed with anti‐GFP, anti‐MYC and anti‐FLAG antibodies. Ponceau S staining (PS) shows equal loading of the samples.

## Data Availability

The data that support the findings of this study are available from the corresponding author upon reasonable request.
