# Supplementary figures and images for: The Recognition of YopJ Family Effectors Depends on ZAR1/JIM2 Immune Complex in Nicotiana benthamiana
Source: Mol Plant Pathol. 2026 Feb 5;27(2):e70214. doi: 10.1111/mpp.70214 (PMC12874497; doi:10.1111/mpp.70214)

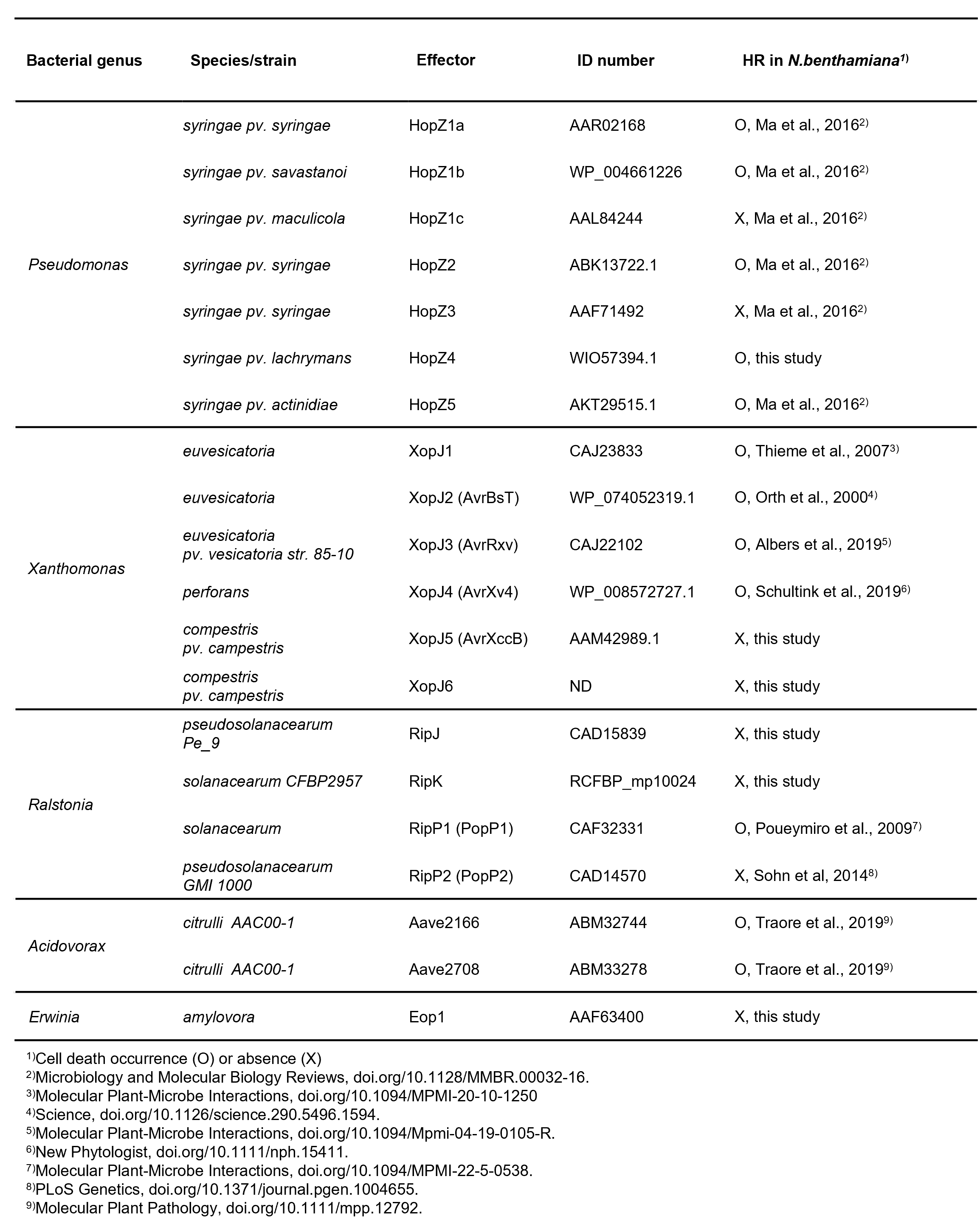

Supplement: Supplementary file 1 — Figure S1: Features of the 20 YopJ family effectors selected for this study. [file MPP-27-e70214-s006.tif]

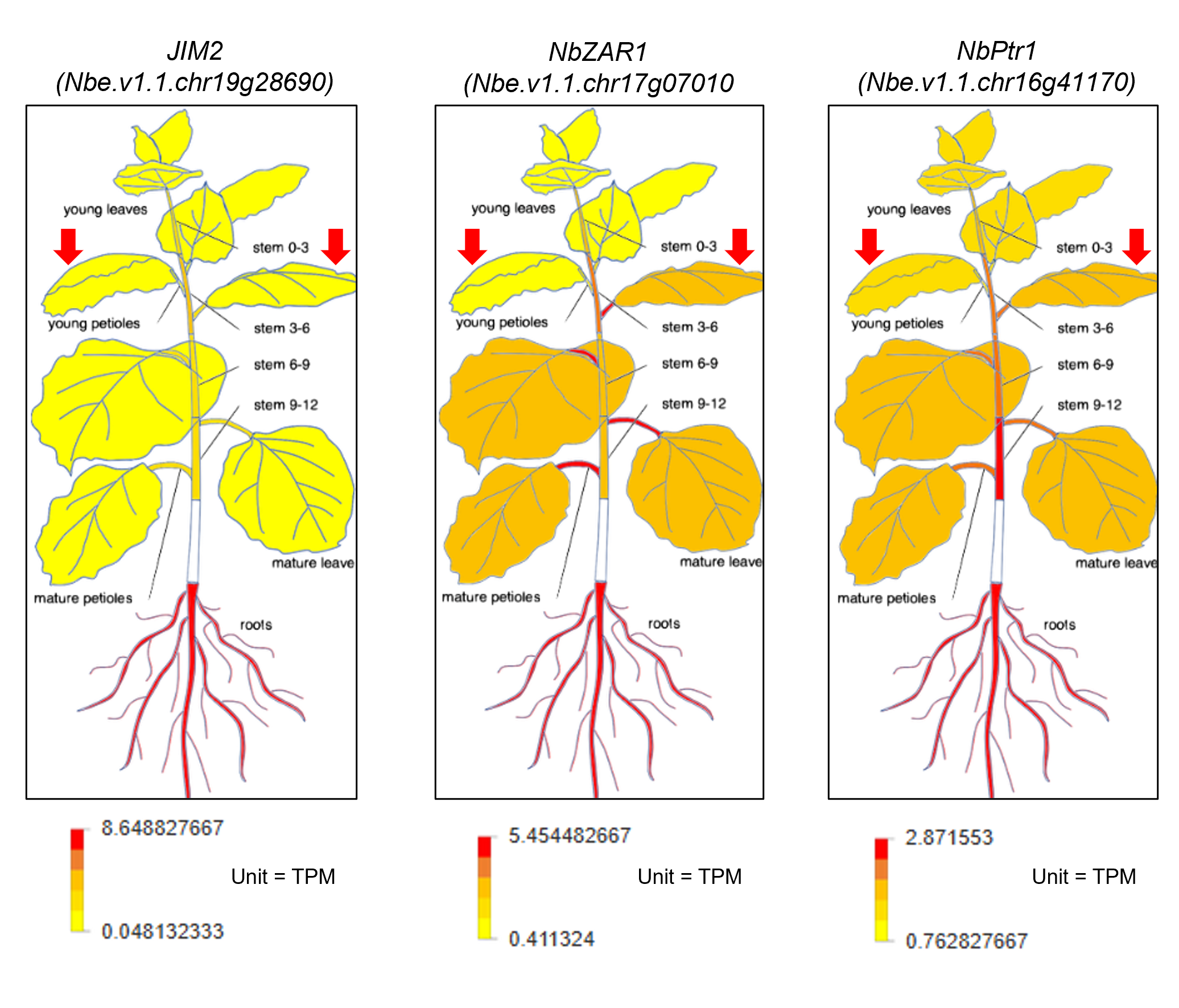

Supplement: Supplementary file 2 — Figure S2: Gene expression profile of JIM2, NbZAR1 and NbPtr1. Expression of JIM2, NbZAR1 and NbPtr1 in transcript per million (TPM) was retrieved on the NbenBase gene expression tool (https://nbenthamiana.jp/nbrowser/profile) (Kurotani et al. 2025). Red arrows indicate leaves used for the agro‐infiltration experiments. [file MPP-27-e70214-s004.tif]

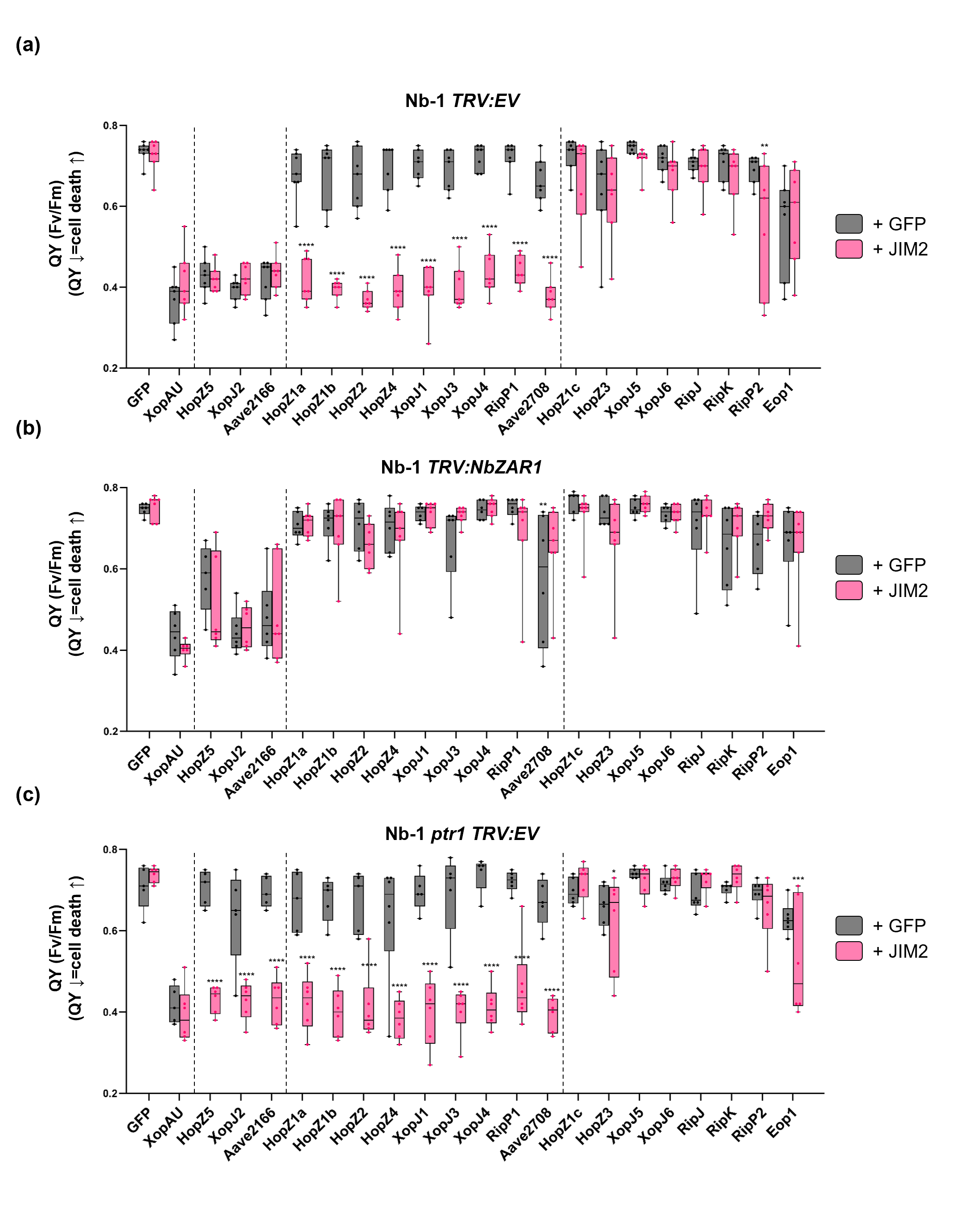

Supplement: Supplementary file 3 — Figure S3: Quantification of cell death in YopJ family T3E expressing tissues shown in Figure 1a. Cell death intensity was quantified by measuring the quantum yield (QY) of each infiltrated spot. High QY indicates strong cell death at the infiltration site, and low QY indicates weak or no cell death. Box plots show the distribution of individual values between the lower and upper quartiles (25%–75%), individual values (dots) and median value (line). The whiskers indicate minimum and maximum values. Statistical comparisons were performed using two‐way ANOVA with JIM2 co‐expression as a main factor and effector identity included as a blocking factor to account for grouped comparisons, followed by Dunnett's multiple comparison test. Asterisks indicate statistically significant differences compared with the GFP control co‐expressed with GFP or JIM2 for each effector in each type of plant (**p < 0.01; ***p < 0.001; ****p < 0.0001). This experiment was independently repeated twice with at least four replicated spots. [file MPP-27-e70214-s001.tif]

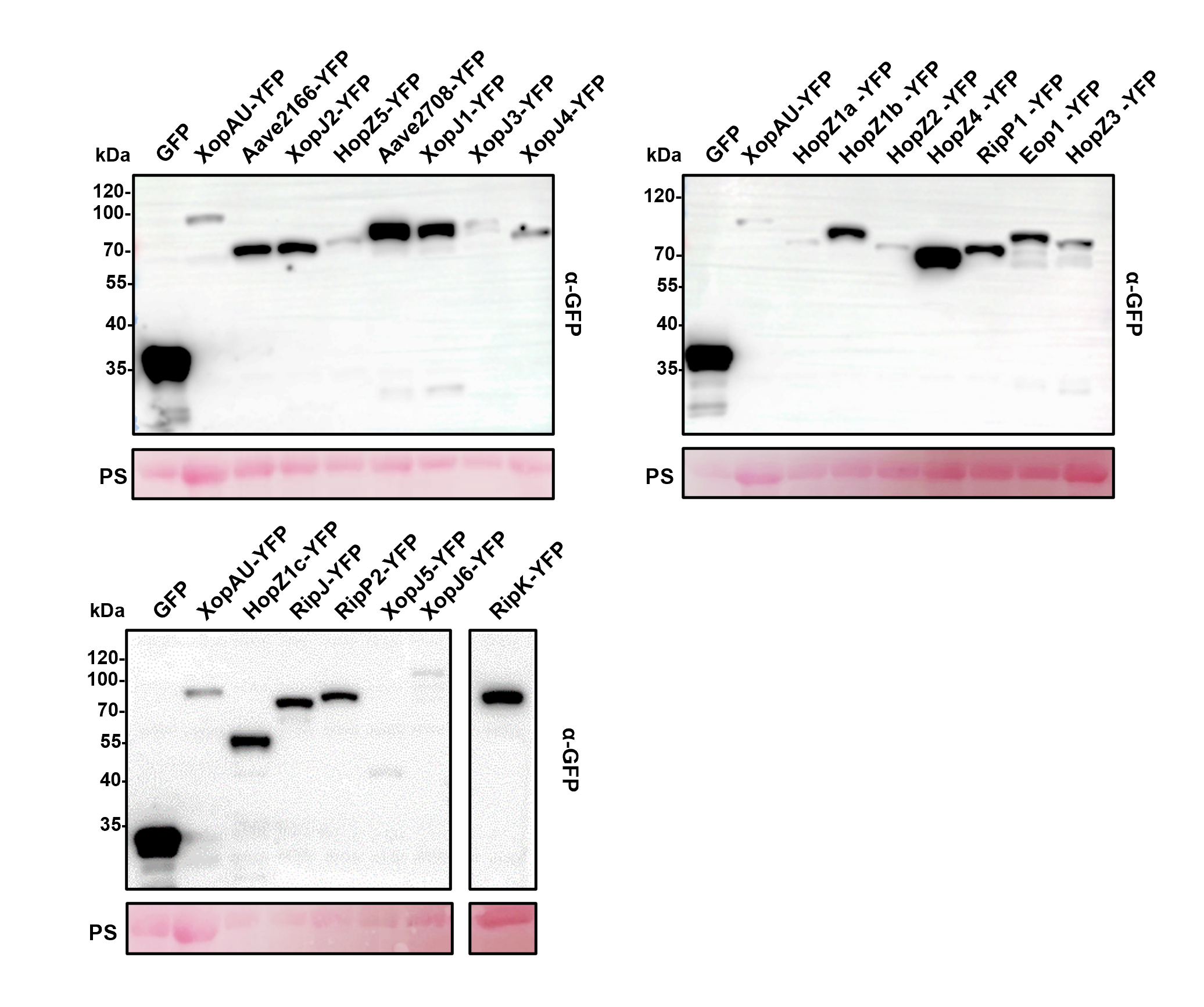

Supplement: Supplementary file 4 — Figure S4: Accumulation of YopJ family T3E proteins in tissues shown in Figure 1. GFP and effector‐YFP fusions were expressed with JIM2‐4xMyc in Nb‐1 ptr1 TRV: NbZAR1 tissues. Leaf samples were harvested 40–60 h after agroinfiltration. Immunodetection on total protein extracts was performed with anti‐GFP antibodies. Ponceau S staining (PS) shows equal loading of the samples. [file MPP-27-e70214-s003.tif]

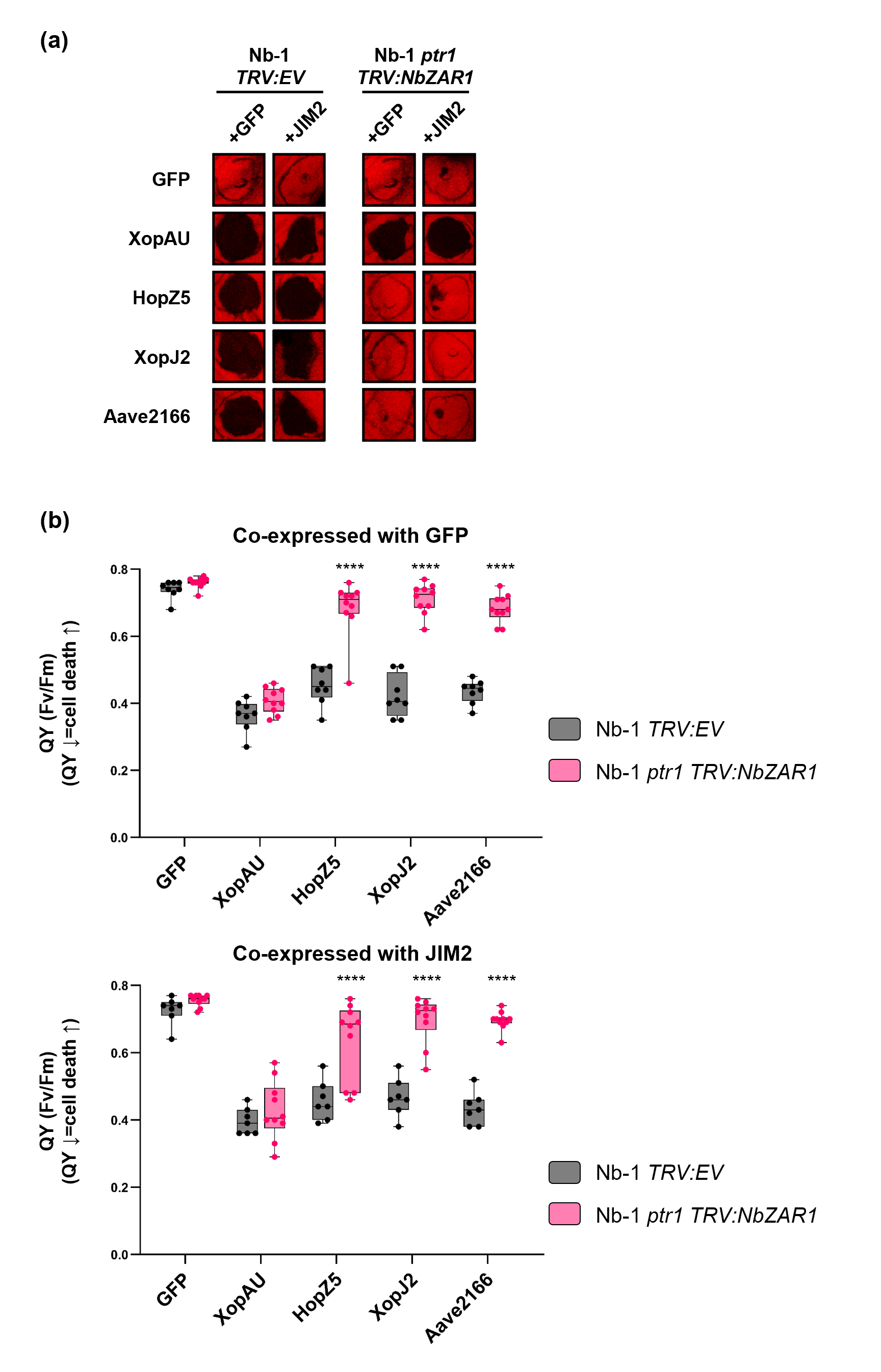

Supplement: Supplementary file 5 — Figure S5: HopZ5, XopJ2 and Aave2166 are independently recognised by NbPtr1 and NbZAR1. (a) GFP, XopAU and each YopJ family effector were co‐expressed with P19 and either GFP or JIM2‐4xMyc in Nb‐1 TRV: EV or in Nb‐1 ptr1 TRV: NbZAR1 silenced plants. (b) Cell death intensity was quantified by measuring the quantum yield (QY) of each infiltrated spot. High QY indicates strong cell death at the infiltration site, and low QY indicates weak or no cell death. Box plots show the distribution of individual values between the lower and upper quartiles (25%–75%), individual values (dots) and median value (line). The whiskers indicate minimum and maximum values. Statistical comparisons were performed using two‐way ANOVA with JIM2 co‐expression and types of plant as main factors and effector identity included as a blocking factor to account for grouped comparisons, followed by Dunnett's multiple comparison test. Asterisks indicate statistically significant differences between Nb‐1 TRV: EV and Nb‐1 ptr1 TRV: NbZAR1 for each effector (****p < 0.0001). This experiment was independently repeated twice with at least 4 replicated spots. [file MPP-27-e70214-s008.tif]

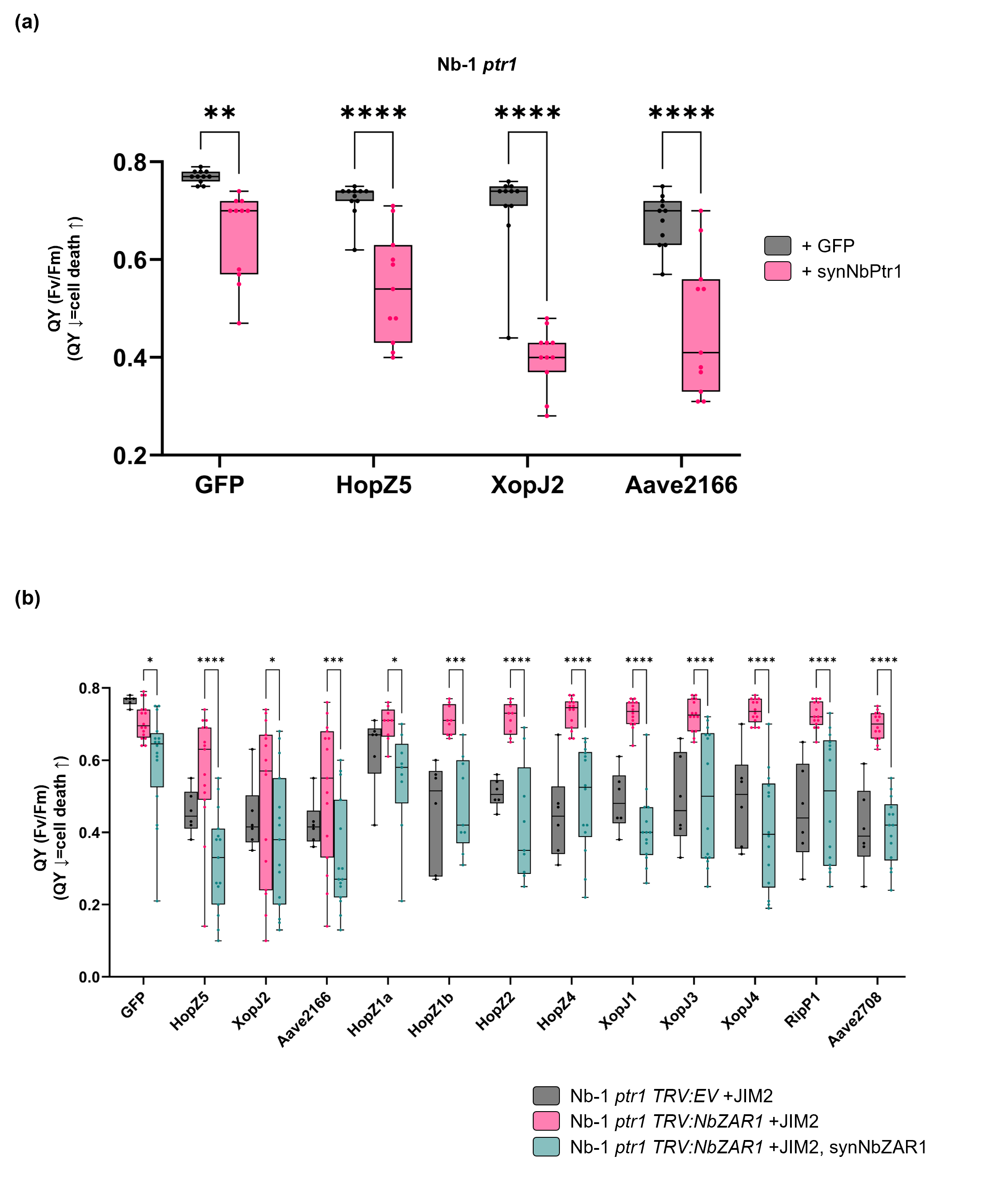

Supplement: Supplementary file 6 — Figure S6: Transient expression of synthetic NbPtr1 and NbZAR1 complements the recognition of YopJ family T3Es in NbPtr1 knock‐out and NbZAR1 knock‐down plants. Cell death intensity was quantified by measuring the quantum yield (QY) of each infiltrated spot. High QY indicates strong cell death at the infiltration site, and low QY indicates weak or no cell death. Box plots show the distribution of individual values between the lower and upper quartiles (25%–75%), individual values (dots) and median value (line). The whiskers indicate minimum and maximum values. Statistical comparisons were performed using two‐way ANOVA with JIM2 co‐expression as a main factor and effector identity included as a blocking factor to account for grouped comparisons, followed by Dunnett's multiple comparison test. These experiments were independently repeated twice with at least four replicated spots. (a) GFP and group I YopJ family effectors (OD600 = 0.5) were co‐expressed with P19 (OD600 = 0.1) and either GFP or synNbPtr1‐6xHA (OD600 = 0.05) in Nb‐1 ptr1 plants. Infiltrated leaves were photographed in light‐emitting diode (LED) light at 3 dpi. Asterisks indicate statistically significant differences between co‐expressed with GFP or synNbPtr1‐6XHA for each effector in each type of plant (**p < 0.01; ****p < 0.0001). (b) GFP, group I and II YopJ family effectors (OD600 = 0.5) were co‐expressed with P19 (OD600 = 0.1), JIM2‐4XMYC (OD600 = 0.05) and either GFP or synNbZAR1‐4XMYC (OD600 = 0.05) in Nb‐1 ptr1 TRV: EV and TRV: NbZAR1 silenced plants. Infiltrated leaves were photographed in light‐emitting diode (LED) light at 2 dpi. Asterisks indicate statistically significant differences of JIM2‐mediated cell death between co‐expression with GFP or synNbZAR1‐4XMYC for each effector. (*p < 0.05; ***p < 0.001; ****p < 0.0001). [file MPP-27-e70214-s010.tif]

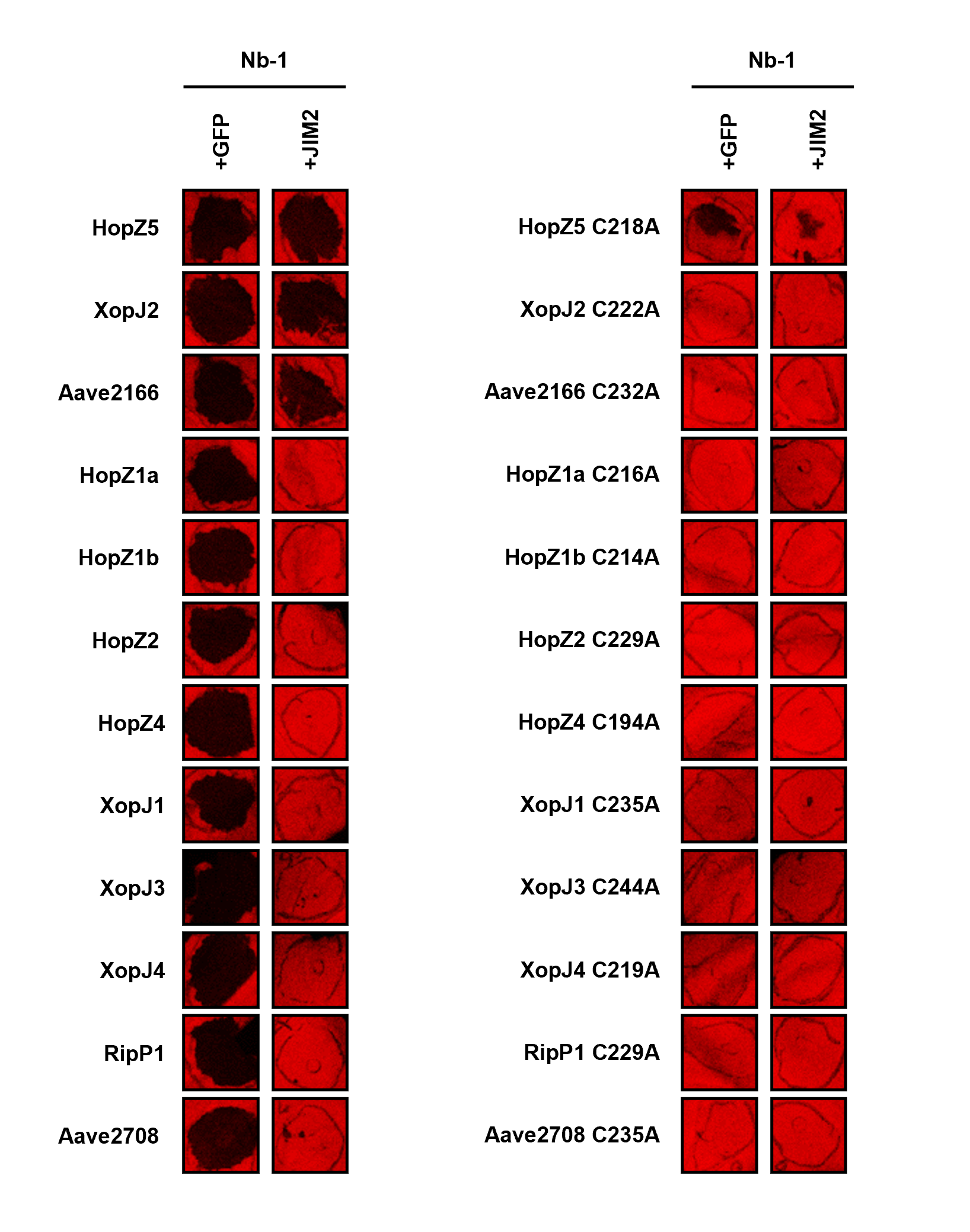

Supplement: Supplementary file 7 — Figure S7: Photographs of infiltrated leaf spots shown in Figure 2. Leaves were photographed in light‐emitting diode (LED) light (red‐orange 617 nm and cool white 6500 K) at 3 dpi. [file MPP-27-e70214-s002.tif]

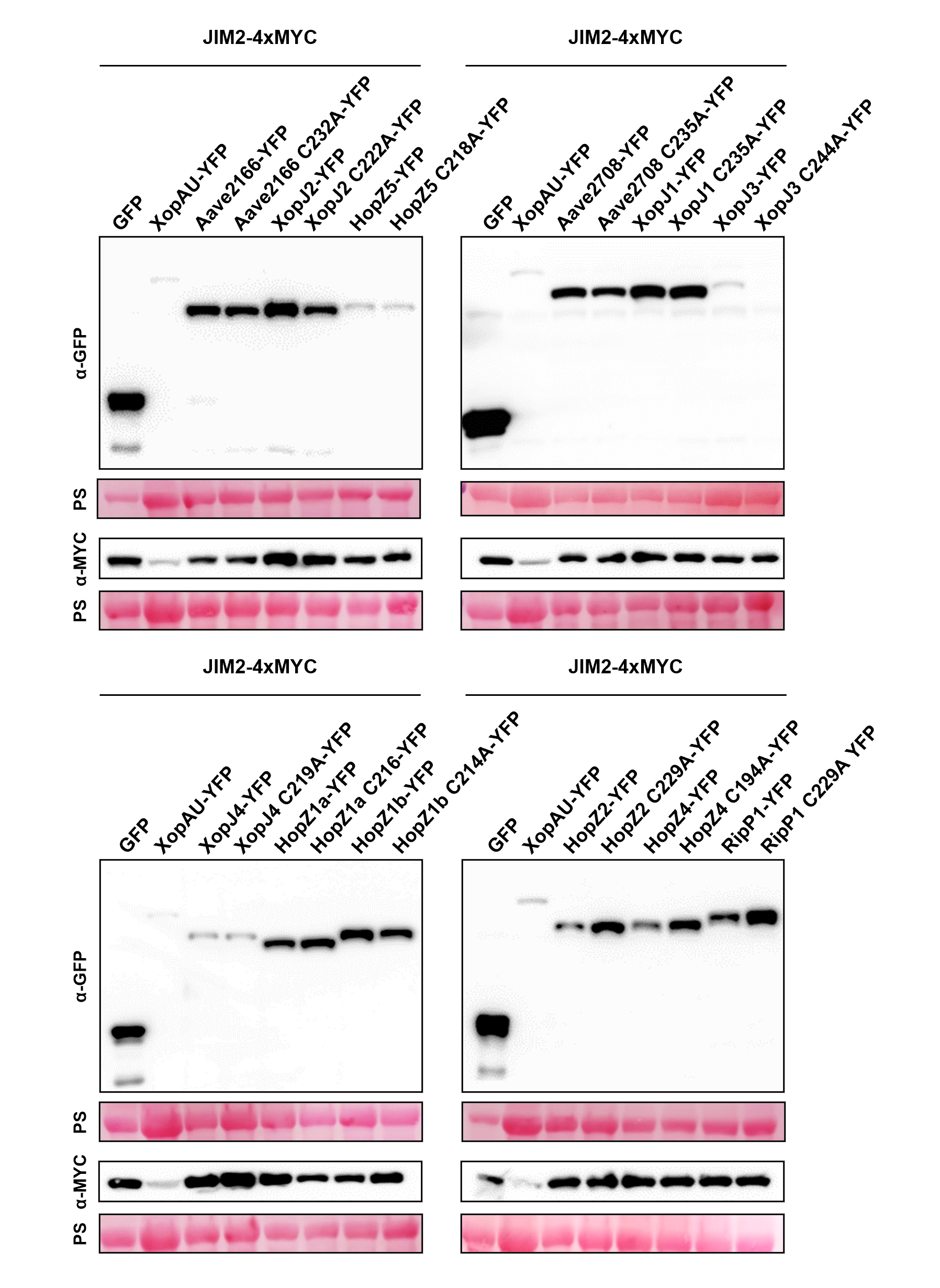

Supplement: Supplementary file 8 — Figure S8: Accumulation of YopJ family T3E C/A mutant proteins in tissues shown in Figure 2. GFP and C/A mutant‐YFP fusions were expressed with JIM2‐4xMyc in Nb‐1 ptr1 TRV: NbZAR1 tissues. Leaf samples were harvested 40–60 h after agroinfiltration. Immunodetection was performed with anti‐GFP and anti‐MYC antibodies. Ponceau S staining (PS) shows equal loading of the samples. [file MPP-27-e70214-s009.tif]

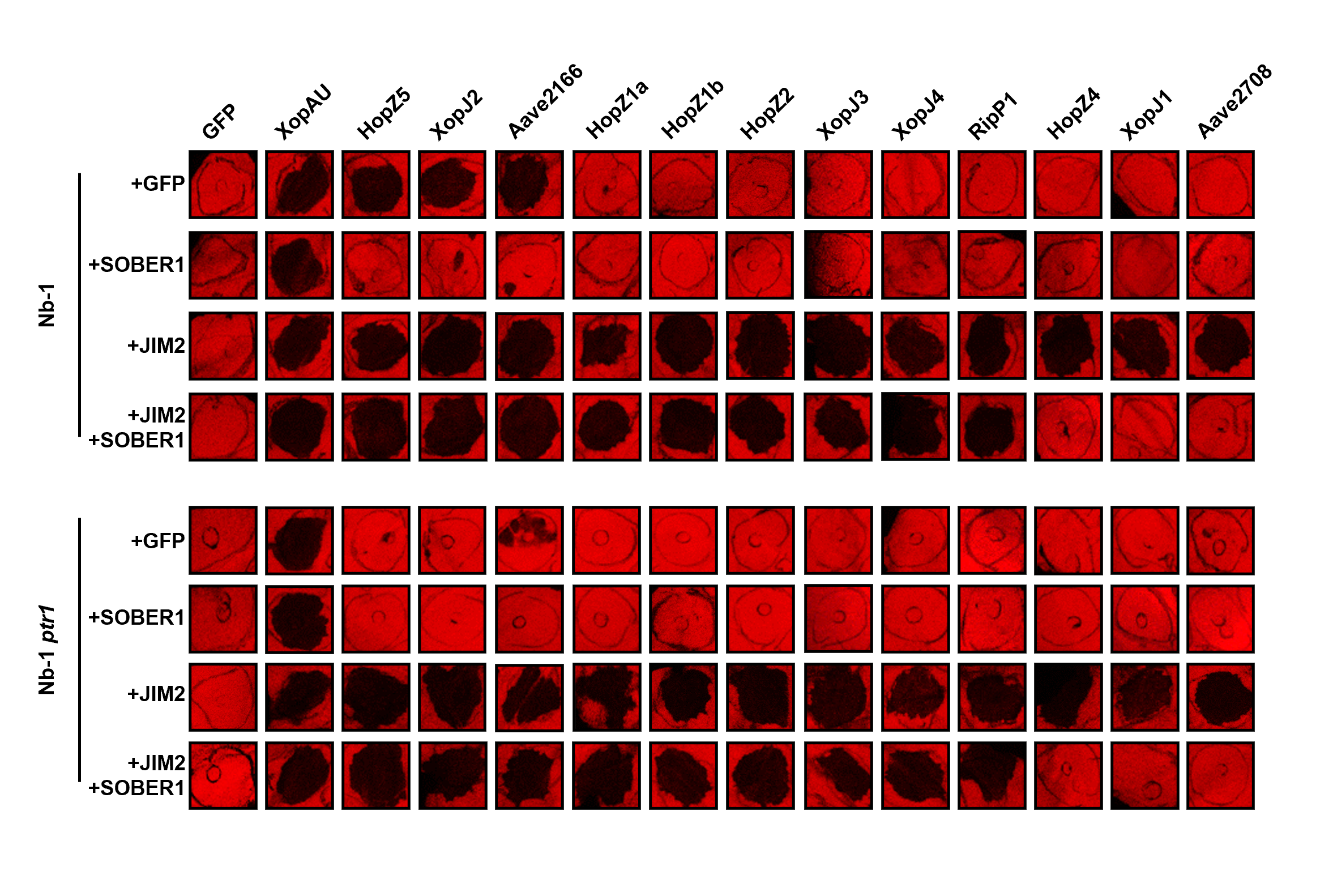

Supplement: Supplementary file 9 — Figure S9: Photographs of infiltrated leaf spots shown in Figure 3. Leaves were photographed in light‐emitting diode (LED) light (red‐orange 617 nm and cool white 6500 K) at 3 dpi. [file MPP-27-e70214-s007.tif]

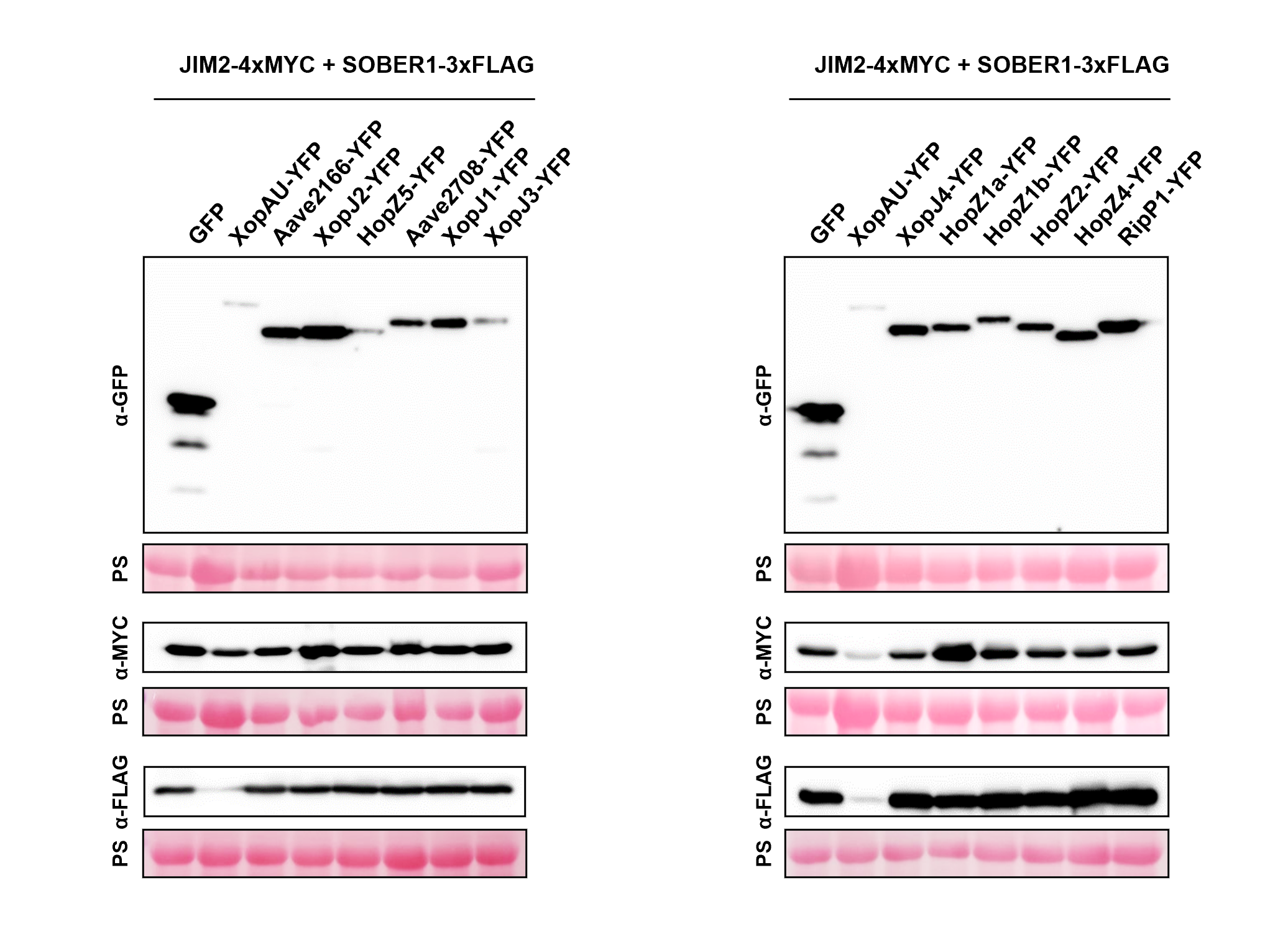

Supplement: Supplementary file 10 — Figure S10: Accumulation of YopJ family T3E, JIM2 and SOBER1 proteins in tissues shown in Figure 3. GFP and effector‐YFP fusions were expressed with JIM2‐4xMyc and SOBER1‐3xFLAG in Nb‐1 ptr1 TRV: NbZAR1 tissues. Leaf samples were harvested 40–60 h after agroinfiltration. Immunodetection was performed with anti‐GFP, anti‐MYC and anti‐FLAG antibodies. Ponceau S staining (PS) shows equal loading of the samples. [file MPP-27-e70214-s005.tif]
